# Studies in Heterogenesis

**Published:** 1904-06

**Authors:** 


### Reviews of Books.

**Studies in Heterogenesis.** By H. CHARLTON BASTIAN, M.D., F.R.S. Pp. ix., 354, with three appendices and many plates containing hundreds of photographic illustrations. London: Williams and Norgate. 1903.

We had thought that this subject had been consigned to oblivion, and that the heterogenetic origin of bacteria and their allies had long ceased to be a serious problem. It is an old story, and one fought out vigorously by the author in opposition to the principal leaders in scientific work. In the life of Pasteur (reviewed in this *Journal*, vol. xx., p. 71) we find the following sentence: "'The confusion and uncertainty,' wrote Tyndall to Pasteur, 'have finally become such that, six months ago, I thought that it would be rendering a service to science, at the same time as justice to yourself, if the question were subjected to a fresh investigation.' . . . Accordingly Tyndall 'went over a large portion of the ground on which Dr. Bastian had taken up his stand, and refuted many of the fallacies which had misled the public.' Pasteur was so anxious to drive Dr. Bastian to the wall for the following reason, which we give in his own words, written to Dr. Bastian in 1877: 'Do you know why I desire so much to fight and conquer you? It is because you are one of the principal adepts of a medical doctrine which I believe to be fatal to progress in the art of healing—the doctrine of the spontaneity of all diseases.'"

It has not yet been forgotten how keenly this conflict was fought, and we were under the impression that Bastian had long since allowed that Pasteur had been the victor.

It comes to us, therefore, as a surprise that Dr. Bastian still clings to his convictions with a doggedness and pertinacity worthy of all commendation in what he believes to be a good cause. He still adheres to the *per saltum* theory of the origin of life, and he still believes in the spontaneity of living organisms. He declines to admit that life is to be defined as a power derived only from pre-existing living beings, and that all organisms—even the most lowly bacteria—breed true. His studies have been laborious and protracted, and this volume is a record of many years of indefatigable labour. The production of the volume is really the work of a lifetime, and as such it demands careful study from those who are qualified to undertake it.

The heterogenetic origin of bacteria and their allies is the subject of section xxxiii., and here we have numerous instances of what appears to be heterogenesis, as shown by the appearance of bacteria in parts of potatoes, apples, oranges, and in the *Cyclops quadricornis*, under circumstances where infection from without does not appear to be possible. So also the author believes that his observations show that the bacteria have in reality been born in the individual cells of the uninfected kidney, and that "the *de novo* origin of bacteria from specks of living matter born directly from certain mother liquids —that is, by a process of synthesis from its elements, and just as crystals are born in other mother liquids"—must still be accepted.

He also raises the question whether heterogenesis is an unknown factor in evolution, and whether it is not a part of and in accordance with the system of nature. He shows "how convertible many of these alien derivates are—how in the pellicle on a hay infusion masses of zooglæa are formed which segment now into fungus germs, now into flagellate monads, and now into amœbæ; and similarly that the substance of a rotifer's egg may under different conditions segment into flagellate monads, amœbæ, paranemata, or even into ciliated infusoria; that on different occasions one or other of the same kinds of organisms may take origin from the substance of large encysted amœbæ; while an encysted ciliate may also itself break up into segments which develop sometimes into flagellate monads, and at others into amœbæ, paranemata, or fungus germs. . . . Finally, I have shown there is no escape from the conclusion that different kinds of bacteria may take their origin within the tissues and cells of animal organisms, and that bacteria and their allies, as well as the simpler forms of fungi, may have a similar *de novo* origin within the closed cells of many vegetables and fruits." The author goes on to remark that "however astounding these statements may seem to those who have not worked long and earnestly at such subjects for themselves, I venture to submit that the facts adduced in this volume, backed by the appearances preserved in the microphotographs, should go far to convince those who will give the subject a fair and impartial consideration."

It is very easy to any critic to make the remark that although Dr. Bastian's plethora of photographic puzzles cannot misrepresent what he saw, he may have himself misrepresented them, and it seems to the reviewer to be more likely that one observer should fail in the discovery of any fallacy in his arguments than that the whole world should have gone wrong on this important question. We must therefore adhere to the view that the evidence is inconclusive, and that some fallacy underlies most of these observations and arguments even if any one individual may fail to find it.

The admission of the author's argument would lead to very serious results in the application of them to medical and surgical questions. He directs attention to this point in his third appendix, an essay of fifteen pages "on the great importance from the point of view of medical science of the proof that bacteria and their allies are capable of arising *de novo*." Here we cannot but believe that Pasteur, although dead, yet speaketh, and his message to us is that the *contagia viva* are not products of spontaneous generation, and that contagious diseases are propagated only and do not arise *de novo*. We are told to "Prove all things: hold fast that which is good." The author is a pioneer in this effort, and we cordially endorse his efforts "to strive to ascertain the conditions of origin of all contagious diseases. The more contagious they are, the more important does the quest become. Let us not blindly think that contagion is the one and only cause, but seek in all doubtful and obscure cases, and by accumulation of evidence, to ascertain what are the invariable and immediately antecedent sets of conditions or states of system that may have sufficed to engender this or that contagious disease." We venture to think that what the author describes as the "narrower ultra-contagionist doctrine" not only is prevailing, but will continue to prevail.

